# Characterization of ES10 lytic bacteriophage isolated from hospital waste against multidrug-resistant uropathogenic *E. coli*

**DOI:** 10.3389/fmicb.2024.1320974

**Published:** 2024-03-08

**Authors:** Aneela Nawaz, Sabeena Zafar, Abdulrahman H. Alessa, Nauman Ahmed Khalid, Muqaddas Shahzadi, Alina Majid, Malik Badshah, Aamer Ali Shah, Samiullah Khan

**Affiliations:** ^1^Department of Microbiology, Faculty of Biological Sciences, Quaid-i-Azam University, Islamabad, Pakistan; ^2^Department of Biology, Faculty of Science, University of Tabuk, Tabuk, Saudi Arabia

**Keywords:** antibiotic resistance, bacteriophage, *E. coli*, phage therapy, biofilm

## Abstract

*Escherichia coli* is the major causative agent of urinary tract infections worldwide and the emergence of multi-drug resistant determinants among clinical isolates necessitates the development of novel therapeutic agents. Lytic bacteriophages efficiently kill specific bacteria and seems promising approach in controlling infections caused by multi-drug resistant pathogens. This study aimed the isolation and detailed characterization of lytic bacteriophage designated as ES10 capable of lysing multidrug-resistant uropathogenic *E. coli*. ES10 had icosahedral head and non-contractile tail and genome size was 48,315 base pairs long encoding 74 proteins. Antibiotics resistance, virulence and lysogenic cycle associated genes were not found in ES10 phage genome. Morphological and whole genome analysis of ES10 phage showed that ES10 is the member of *Drexlerviridae*. Latent time of ES10 was 30 min, burst size was 90, and optimal multiplicity of infection was 1. ES10 was stable in human blood and subsequently caused 99.34% reduction of host bacteria. Calcium chloride shortened the adsorption time and latency period of ES10 and significantly inhibited biofilm formation of host bacteria. ES10 caused 99.84% reduction of host bacteria from contaminated fomites. ES10 phage possesses potential to be utilized in standard phage therapy.

## 1 Introduction

Antibiotics are used in healthcare system for therapeutic purposes, in food industry, agriculture, and veterinary practices for decades, but the indiscriminate use of antibiotics has led to an increase in multi-resistant pathogens (Tiedje et al., 2023). Due to the decline in research and development of new antibiotics against resistant bacterial pathogens, very few antibiotics are effective against pathogenic bacteria (Tacconelli et al., 2018). Multi-drug resistant bacteria are the major threat to public health and is considered as the major problem of this era. Antibiotic resistance has become a worsening crisis worldwide and represents the greatest burden on health and the economy (Founou et al., 2021). World Health Organization has declared that antimicrobial resistance is among top ten global health threats (Walsh et al., 2023). The rise in antimicrobial resistance is diminishing the efficacy of currently available antibiotics that were being used for treating widespread bacterial infections. Global Antimicrobial Resistance and Surveillance System in 2022 had reported that the emergence of antimicrobial resistance in most prevalent bacterial pathogens is alarming (Zaghen et al., 2023). EARS-Net has reported that 670,000 infections and 33,000 deaths caused by multi-drug resistant pathogens, occurs per annum in Europe (Gabutti, 2022). In United States, more than 2.8 million infections caused by multi-drug resistant pathogens occur annually (Morris and Cerceo, 2020). Antimicrobial resistant pathogens are associated with 1.27 million deaths worldwide (Salam et al., 2023). According to World Health Organization, 45% death in South East-Asia is due to infections caused by multi-drug resistant pathogens (Chen et al., 2023). Prevalence of multi-drug resistant pathogen is high in low-and middle income countries compared to high income countries (Algammal et al., 2023). Pakistan is ranked third for antibiotics consumption in low and middle income countries that has exacerbated emergence of antimicrobial resistant strains (Torumkuney et al., 2022). By 2050, 10 million deaths per year are expected to be caused by infections caused by multidrug-resistant pathogens (Painuli et al., 2023).

Urinary tract infections are the most common infections worldwide and 150 million cases occur worldwide, annually (Zeng et al., 2022). Mortalities associated with urinary tract infections in elderly patients are 6.2% (Gharbi et al., 2019). *E. coli* is the common cause of community-acquired (80%−90%) and hospital-acquired (30%−50%) urinary tract infections (Ramírez-Castillo et al., 2018). Uropathogenic *E. coli* is the predominant pathogen harboring various virulence factors (fimbriae, adhesins, and siderophore aerobactin) involved in causing infections (Behzadi and Behzadi, 2017) and protecting it from the host immune system (Sarowska et al., 2019). Traditional antibiotics have been prescribed to treat urinary tract infections caused by *E. coli*, but antibiotics have become a less acceptable choice due to increasing resistance. The common mechanisms for acquiring resistance to now available antibiotics in *E. coli* is the development of extended spectrum beta lactamases, the CTX-M enzyme responsible for resistance to fluoroquinolones, and efflux pumps (Lepe and Martínez-Martínez, 2022).

Increasing antibiotic resistance has led researchers to look for alternative treatment to cure infections. Lytic bacteriophages, natural antibacterial agents are capable of lysing multidrug-resistant pathogens as the mechanisms involved in the emergence of antibiotic resistance are different from the mechanisms that bacteriophages use to lyse host bacteria (Taati Moghadam et al., 2020). Phage therapy is fundamentally different from conventional antibiotics. Phages reproduce naturally in the host bacteria after infecting it and cause the host cell to lyse for progeny release. Due to the specificity of the phages toward the host, smaller amounts of endotoxins are released after the bacterial cells are lysed. Phages are also capable of degrading biofilm (Hanlon, 2007). Compared to broad-spectrum antibiotics, phages are less likely to disrupt human intestinal flora (Lin et al., 2017).

In previous study, urinary tract infection caused by uropathogenic *E. coli* in mice model was treated with T4 and KEP10 phages. In 90% of the infected mice were saved from death by treatment with these phages compared to untreated mice that died within 3 days of infections (Nishikawa et al., 2008). myPSH1131 bacteriophage has been reported previously, this phage was capable of lysing 31 pathogenic strains of *E. coli*. Single dose of myPSH1131 has increased the life span of infected larva of *G. mellonella* from 24 to 48 h, and administration of three to four doses of this phage has reduced mortality rate to zero that confirmed the eradication of pathogenic *E. coli* (Alexyuk et al., 2022). Millions of people are succumbed to infections caused by multi-drug resistant pathogens, worldwide, based on previous studies on phage therapy, the utilization of phage therapy alternative to antibiotics can be a viable approach in treating multi-drug resistant pathogens.

The aim of this study was to isolate and characterize lytic bacteriophage ES10 effective against uropathogenic *E. coli*. In this study, the physiochemical characterization of lytic phages, stability and efficacy in human blood, and efficacy on contaminated surfaces were evaluated and presented.

## 2 Materials and methods


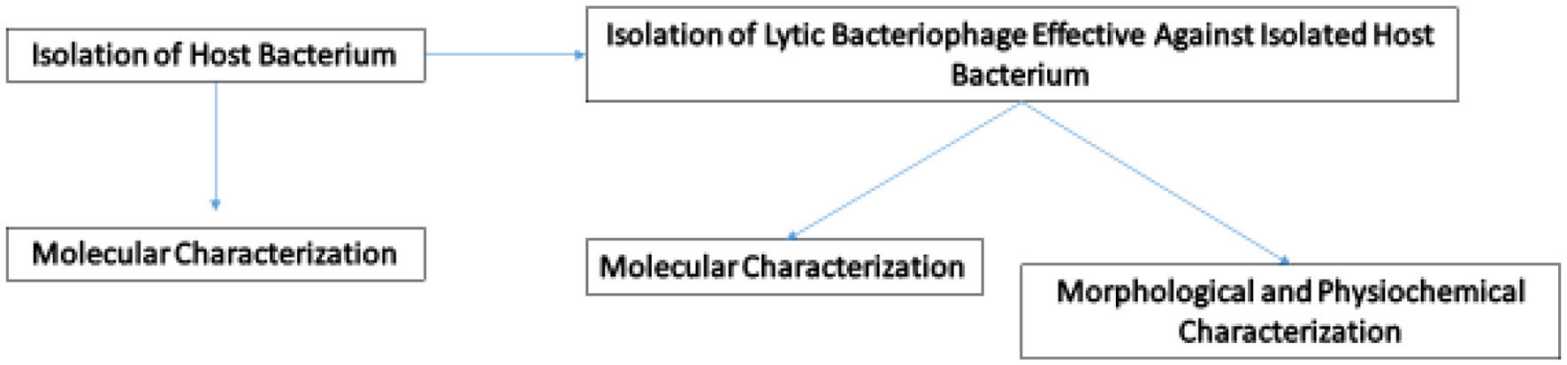


### 2.1 Location of study

The present study was conducted in the Laboratory of Applied Environmental and Geomicrobiology (AEG), Department of Microbiology, Quaid-i-Azam University, Islamabad, Pakistan.

### 2.2 Isolation and characterization of host bacteria

The bacterial strain isolated from the urine sample of a 68-year-old woman with urinary tract infection was streaked in quadrants on eosin-methylene blue agar (70186 EMB agar, MilliporeSigma), and colony morphology and green sheen was observed after 24 h of incubation at 37°C.

*In vitro* and *in silico* characterization of this isolated uropathogenic *E. coli* was done in this study (Behzadi and Ranjbar, 2019). Antibiotic susceptibility test of this strain against amoxicillin (AML-10), bacitracin (B-10), cefixime (CFM-5), cefotaxime (CTX-30), ceftazidime (CAZ-30), clindamycin (CD-10), fusidic acid (FD-10), oxacillin (OX-1), pipracillin (30 g), gentamicin (30 g), and meropenem (10 g) were performed by Kirby-Bauer disk diffusion assay (Yang et al., 2019) according to Clinical and Laboratory Standard Institute (CLSI) guidelines. The disks used in this study were purchased from Oxoid ThermoFisher Scientific, United Kingdom.

The biofilm production potential of this strain was checked using the microplate method described previously (Parasana et al., 2023) with some modifications, briefly, 24 h old broth culture of host bacteria was diluted in nutrient broth and 100 μL of diluted culture was added in wells of flat bottom polystyrene microtiter plate and incubated at 37°C for 24 h. After incubation, wells were washed thrice with distill water and 125 μL of 0.1% crystal violet was added in each well and incubated for 15 min at room temperature. After incubation, microtiter plate was washed thrice to remove excess dye. In 125 μL of 30% acetic acid was added in each well to dissolve crystal violet and incubation of 15 min at room temperature was given. After incubation, solubilized crystal violet was added in wells of new microtiter plate and OD at 570 nm was checked and 30% acetic acid was used as negative control. Biofilm formation potential was calculated using the following formula used previously (Singh et al., 2017).

For genomic DNA extraction of the host bacteria, 2 mL of a 24-h-old host bacterial broth culture (OD_600_ 0.959) was centrifuged at 10,000 rpm for 2 min, the supernatant was discarded and the pellet was immersed in 200 μL of cold phosphate buffer saline (NaCl 137 mM, KCl 2 mM, Na2HPO4 10 mM, and KH2PO4 1.8 mM) and genomic DNA were extracted using the kit (Monarch Genomic DNA Purification Kit, BioLabs, New England with catalog number T3010S) according to the protocol provided with the kit. The integrity of the extracted DNA was assessed by gel electrophoresis (0.7% agrose), and DNA quantification and purity were assessed by Nanodrop. This extracted DNA was sent to SeqCenter, Pittsburgh, PA 15201, USA for Illumina whole genome sequencing. Paired end reads obtained in the Fastq file with 100x coverage were assembled online at https://www.bv-brc.org/app/Assembly2 and the SPAdes assembly strategy was selected. Host bacterial DNA assembled into contigs was submitted to the National Center for Biotechnology Information (NCBI) (https://submit.ncbi.nlm.nih.gov/subs/genome/) for annotation and accession number, as well as comprehensive genomic analysis of the assembled contigs were carried out online at https://www.bv-brc.org/app/ComprehensiveGenomeAnalysis to analyze antibiotics resistance genes.

### 2.3 Sample collection

Waste water sample was collected from King Abdullah Teaching Hospital, located in Mansehra Khyber Pakhtunkhwa, Pakistan, for bacteriophage isolation in 50 mL falcon tubes. The sampling areas were specifically selected based on the feasibility of the available wastewater.

### 2.4 Bacteriophage isolation and purification

The collected sample was processed for isolation of bacteriophages using the previously described protocol (Kumari et al., 2010) with some modifications. Briefly, 20 mL of the sample was centrifuged at 10,000 rpm for 5 min and filtered through a 0.22 μm syringe filter and mixed with 20 mL of 2X nutrient broth and 1 mL of a 24-h-old host bacterial culture and incubated overnight at 37°C. The host bacteria-enriched samples were centrifuged at 10,000 rpm for 5 min, and the supernatant was filtered through a 0.2 μm pore size syringe filter and assayed for the presence of bacteriophages using the spot assay described previously (Nabil et al., 2018), briefly, 10 μL of phage filtrate spotted onto a nutrient agar plate inoculated with 100 μL of host bacteria (OD600 0.5) in a 0.7% soft agar layer. After the observation of clear spot, phage filtrate was serially diluted and subjected to a double-layer agar assay described previously (Santos et al., 2009) to obtain well-isolated and clear plaques. In double layer agar assay, 100 μL of dilutions of filtrate were mixed with 100 μL of 24 h old host bacterium culture, and phages were allowed to adsorb on host bacterium for 10 min, then, 3 mL of 0.7% soft agar was added in it and poured on freshly prepared plates of nutrient agar. Plates were incubated at 37°C for 24 h. After incubation, plaques were visualized. The single clear plaque was purified three times by double layer agar assay.

### 2.5 Bacteriophage titer and stability determination in glycerol stock

The bacteriophage titer was increased by inoculating approximately 1 mL of phage filtrate in 10 mL of logarithmic phase bacterial culture and incubated for 8 h in a shaking incubator at 37°C. After incubation, 5 mL sample was centrifuged, filtered through 0.22 μm membrane syringe filter, 100 μL of phage filtrate was diluted and subjected to double layer agar assay. The clear and well-isolated plaques were counted and expressed as PFU/mL using the formula n × 10 × d, where n is the number of plaques and d is the reciprocal of the dilution factor. After titer determination, the phage filtrate was preserved in triplicate in 100% glycerol at a volume of 1:1 in cryovials and stored at −80°C. 100 μL samples were taken every 3 months for 1 year, serially diluted and checked for stability of ES10 using the double layer agar assay.

### 2.6 Characterization of ES10

#### 2.6.1 Dry lab

Genomic DNA of ES10 phage was extracted using the phenol-chloroform method (Kunisch et al., 2023) from 10 mL of ES10 phage filtrate (2.2 × 10^11^ PFU/mL) treated with DNase I and RNase. Quantification and purification of the extracted DNA was carried out using Nanodrop and the integrity of the extracted DNA was confirmed by gel electrophoresis. The extracted DNA was sent to SeqCenter, Pittsburgh, USA, for whole-genome sequencing by Illumina. Paired-end reads in the Fastq file obtained from SeqCenter were assembled online at BV-BRC (https://www.bv-brc.org/) using the SPAdes assembly strategy. The ES10 phage genome contig was annotated on BV-BRC (https://www.bv-brc.org/app/Annotation), PHASTER and DFAST v1.2.0 (https://dfast.ddbj.nig.ac.jp/). The assembled ES10 phage genome was run on PHASTER (https://phaster.ca/) to find out the completeness of the phage genome. The ES10 phage genome was run on Proksee (https://proksee.ca/projects/new) to identify antibiotic resistance genes by running CARD Resistance Gene Identifier. To predict the therapeutic stability of ES10 phage, the phage genome was run on PhageLeads (https://phageleads.dk/), which runs in parallel in database map, vfdb and Resfinder to find antibiotic resistance genes, virulence genes and the temperate lifestyle genes. The phage genome was run on Bacphlip (https://cpt.tamu.edu/galaxy-pub) to assess the virulent and temperate nature of the ES10 phage. The ES10 phage genome was scanned for tRNA prediction (tRNAscan) by selecting a general tRNA model at https://cpt.tamu.edu/galaxy-pub. Phage promoters were predicted on PhagePromoter (https://galaxy.bio.di.uminho.pt/). A phylogenetic tree based on the similarity of the entire genome sequence of ES10 was constructed on NCBI. The entire genome of ES10 was run on BLASTN. For the phylogenetic tree view (neighbor joining tree and maximum sequence difference of 0.75) on NCBI, whole-genome sequences with query coverage with the ES10 genome of more than 40% and percent identity of more than 75% were selected. The proteomics-based phylogenetic tree of ES10 was constructed on VipTree (https://www.genome.jp/viptree/upload) by analyzing the ES10 phage genome with reference proteome via tBLASTx and GHOSTX. The entire genome of the ES10 phage was submitted to the NCBI for deposit of an accession number.

#### 2.6.2 Wet lab experiments

##### 2.6.2.1 Morphological characterization of ES10 by transmission electron microscopy

The phage filtrate of ES10 with the PFU of 6.8 × 10^9^/mL was diluted 1:10 in normal saline (0.95% NaCl in distilled water) and sent to the National Institute of Biotechnology and Genetic Engineering (NIBGE), Faisalabad, for transmission electron microscopy (TEM). In 100 μL of phage filtrate was spotted onto a 300-mesh copper grid, stained with 2% uranyl acetate, and immediately blotted with filter paper. The sample was air dried and visualized under an electron microscope (EM 10 C supplied by Carl Zeiss, Germany). The length and width of the phage capsid and tail were measured online via Image J (https://ij.imjoy.io/) using the scale bar.

##### 2.6.2.2 Effect of different media, soft agar concentration and incubation time on ES10 plaques morphology

A double layer agar assay of dilutions of ES10 phage filtrate was performed to study how media composition influences plaque morphology. To perform a double layer agar assay, six different media were used: nutrient agar, tryptic soy agar, Luria-Bertani agar, Mueller-Hinton agar, MacConkey agar, and eosin-methylene blue agar as bottom agar with 0.7% top agar. Briefly, 100 μL of dilutions of phage filtrate with 100 μL of host bacteria culture were added with 3 mL of 0.7% soft agar and poured on solidified pre-prepared plates of selected bacteriological media. All plates were incubated at 37°C for 24 h. After incubation, the plaque morphology of the same dilution of ES10 phage filtrate was observed on different media and the plaque size of 10 random plaques was measured and the average plaque size was calculated by dividing the sum of all observations by the number of observations (Ramesh et al., 2019).

Concentration of soft agar used in top agar layer in double layer agar assay effects plaques morphology by effecting the diffusion of bacteriophages across the top layer to target its host bacteria. The effect of different concentrations of soft agar on plaque morphology of ES10 was assessed using a double-layer agar assay of dilutions of ES10 phage filtrate with different concentrations of soft agar (0.5%, 0.7%, 1%, 1.2%, 1.5%, and 2%) used as top agar and nutrient agar was used as bottom agar.

Bacteriophages replicate on solid media inoculated with host bacteria ultimately increase their number and effect neighboring host bacteria that cause increase in plaque size with increase in incubation period. To evaluate the effect of incubation period on plaque size of ES10 phage, isolated plaques of ES10 were observed for 72 h and after every 24 h, plaque size was measured.

##### 2.6.2.3 Thermal, and pH stability

ES10 phage thermal and pH stability assays were performed according to previously described protocols with minor modifications. The filtrate of ES10 phage was incubated at different temperature ranges (0°C, 4°C, 25°C, 30°C, 35°C, 40°C, 45°C, and 50°C) for 4 h and the stability of ES10 phage was assessed by double layer agar assay followed by dilutions (Yang et al., 2010). Similarly, the pH stability of phage ES10 was determined by diluting the ES10 phage filtrate in normal saline with different pH ranges (2, 3, 4, 5, 6, 7, 8, 9, and 10) and incubated for 4 h at 35°C (Abdelghafar et al., 2023). Phage stability was assessed using a double-layer agar assay.

##### 2.6.2.4 Optimal multiplicity of infection

Multiplicity of infection (MOI) is defined as the number of viron particles required per cell during infection. To determine the ideal MOI of the ES10 phage, 1 mL of *E. coli* in logarithmic phase (CFU 2.5 × 10^7^/mL) was mixed with 1 mL of different MOIs (10, 1, 0.1, and 0.01) of the ES10 phage filtrate in separate 100 mL broths, and incubated at 37°C for 24 h. A 1 mL sample was drawn from each flask, centrifuged, filtered, serially diluted, and the dilutions were subjected to a double-layer agar assay. Based on the phage titer, the optimal multiplicity of infection was evaluated (Qu et al., 2023).

##### 2.6.2.5 One step growth

The number of phages required to infect a single bacteria, the latency time, and the burst size of the ES10 phage were determined by performing a one-step growth experiment using the previously published protocol (Kropinski, 2018) with some modifications. One mL of a 24-h-old *E. coli* culture was inoculated into 50 mL of nutrient broth and incubated for 2–3 h until the mid-exponential phase was reached and CFU of this broth culture was determined by spreading it on MacConkey agar followed by dilutions. One mL of culture was pelleted and the pellet was combined by vortexing with 1 mL of nutrient broth and 1 mL of ES10 phage filtrate (4.6 × 10^10^ PFU/mL) and the phages were allowed to adsorb for 10 min. The remaining unabsorbed phages were removed by centrifugation and the number of free phages in the supernatant was determined and the pellet was suspended in 100 mL of nutrient broth and 1 mL of sample is drawn to find out the number of uninfected bacterial cells while rest of the sample was incubated at 37°C for 90 min and after every 10 min 1 mL of the sample was drawn and subjected to plaque assay followed by dilutions to estimate titer of free phages.

The average number of phages infecting a single bacterium was determined using formula.

Number of phages infecting a bacterium = initial PFU – unabsorbed phages/number of infected host bacteria – number of uninfected host bacteria

The burst size was calculated using the formulas:

Burst size = final PFU/number of infected bacteria.

##### 2.6.2.6 Effect of salts on the replication cycle of ES10

The adsorption potential, latency and burst size of ES10 phage in the presence of calcium and magnesium ions were determined according to the previously described protocol. Nutrient broth (100 mL), nutrient broth (100 mL) augmented with 10 mM calcium chloride and nutrient broth (100 mL) augmented with 10 mM magnesium chloride were inoculated with 100 μL of host bacteria (2 × 10^5^ CFU/mL) and 100 μL of phage filtrate (2.2 × 10^6^ PFU/mL). These flasks were kept in shaking incubator (180 rpm) at 37°C and samples were collected from each flask after 10 min intervals for 60 min and subjected to double layer agar assay followed by dilutions, to evaluate the number of free phages (Cobian-Guemes et al., 2023).

##### 2.6.2.7 Effects of salts on turbidity reduction and biofilm formation inhibition of host bacteria by ES10

Nutrient broth, nutrient broth with calcium chloride (10 mmol), and nutrient broth with magnesium chloride (10 mmol) were inoculated with 1 mL of host bacteria and 1 mL of ES10 phage filtrate and incubated for 24 h at 37°C and nutrient broth inoculated with host bacteria was used as a control. After incubation, 1 mL of the samples was drawn, the OD at 600 nm was checked and compared with the OD of the control.

The biofilm inhibition potential of ES10 phage and in combination with salts was evaluated. Three wells with 100 μL of bacterial culture were added with 200 μL of freshly prepared nutrient broth and kept as controls, three wells with 300 μL of nutrient broth were maintained as negative controls, three wells with 100 μL of host bacterial culture were added with 100 μL of nutrient broth and 100 μL of ES10 phage filtrate, three wells with 100 μL of bacterial culture were added 100 μL of ES10 phage filtrate and 100 μL of nutrient broth augmented with 30 mM CaCl2 (final concentration of 10 mM), and three wells with 100 μL of bacterial culture were added 100 μL of ES10 phage filtrate and 100 μL of nutrient broth augmented with 30 mM MgCl2 (final concentration 10 mmol). This micro titer plate was incubated in a static incubator at 37°C for 48 h. After incubation, all wells were washed three times with distill water, air dried and stained with 0.1% crystal violet solution. After 15 min of staining, microtiter plate was washed and air dried and 30% acetic acid was used to dissolve crystal violet and OD at 570 nm was checked and compared with the OD of control.

##### 2.6.2.8 Effect of incubation period, temperature, pH and media on host bacteria turbidity reduction by ES10

The bacterial turbidity reduction assay was performed to determine the optimal time for maximum host bacterial reduction. One mL of host bacterial culture (OD600 1.29) was inoculated into 6 flasks with 100 mL nutrient broth. Three flasks of these were inoculated with 1 mL ES10 phage at MOI 10 and the other flasks were kept as a control without phage filtrate and incubated at 37°C with constant shaking for 2 h. The samples were taken after every 20 min for 2 h and the OD was checked at a wavelength of 600 nm to evaluate optimal incubation period for maximum reduction of host bacterium (Rasool et al., 2016).

The optimal temperature for maximum turbidity reduction of the host bacteria by ES10 phages was analyzed by incubating nutrient broths inoculated with host bacteria and ES10 phage filtrate (MOI 10) at different temperatures (25, 30, 35, and 40°C) for optimal incubation time. The reduction in turbidity was assessed by measuring OD at 600 nm and compared to the control of each temperature (Ameh, 2023). Similarly, the optimal pH for maximum reduction of the host bacteria by the ES10 phage was analyzed. Nutrient broth with different pH (5, 6, 7, 8, and 9) adjusted were inoculated with host bacterium and ES10 phage filtrate (MOI 10) and incubated at optimal temperature for optimal time. After incubation 1 mL samples were drawn from each flask and OD at 600 nm was checked and compared with the OD of control of each pH.

The media used in this experiment were nutrient broth, tryptic soya broth, Luria broth and Muller Hinton broth with optimally adjusted pH. Three sterile broths (100 mL) were prepared for each medium, one broth of each medium was inoculated with 1 mL of host bacteria, and the remaining flasks were inoculated with 1 mL of host bacteria and 1 mL of ES10 phage filtrate (MOI 10). All flasks were incubated at optimal temperature for optimal incubation time and the OD was measured at a wavelength of 600 nm and compared with the control.

##### 2.6.2.9 Stability of ES10 in human blood

To evaluate the effect of plasma proteases on the stability of ES10, *in vitro*, 900 μL of human blood collected in a vacutainer (containing EDTA) was inoculated with 100 μL of ES10 phage filtrate and incubated for 1 h. In 100 μL of ES10 phage filtrate diluted in normal saline in 1:10 was used as control. After incubation, 100 μL of the sample was serially diluted and subjected to a double-layer agar assay. PFU of the phage filtrate inoculated in human blood was compared with the PFU of control.

##### 2.6.2.10 Reduction of host bacteria by ES10 in human blood

To evaluate the efficacy of ES10 phage to lyse host bacteria in human blood, *in vitro*, 990 μL of human blood was inoculated with 10 μL of the host bacteria with a CFU of 1.476 × 10^6^/mL as a control, 990 μL of blood was inoculated with 10 μL of normal saline and kept as a negative control, and 980 μL of human blood was inoculated with 10 μL host bacteria and 10 μL of phage filtrate (MOI 10). After incubation, CFU was determined by spreading 100 μL of the samples followed by serial dilutions on freshly prepared MacConkey agar plates. The reduction in CFU of phage treated human blood was compared with untreated human blood and percentage of reduction in CFU was calculated (Frati et al., 2019).

##### 2.6.2.11 Fomite decontamination assay

The fomite decontamination assay was performed using glass slides to examine the phage's ability to eradicate *E. coli* from contaminated surfaces. Approximately 100 μL of freshly prepared bacterial culture was spread on slides and then allowed to dry, three slides were kept as a control and 100 μL of phage lysate with different MOIs (10, 1, 0.1, and 0.01) was spread on slides and incubated for 1 h. Each slide was placed in a sterile 50 mL flask with broth medium and placed in a shaking incubator for 5 min to dislodge any remaining bacteria on the surface. Ten μL of the sample taken from each broth was spread onto MacConkey agar plates and CFU was found out after 24 h of incubation at 37°C. The percentage of reduction in CFU of host bacteria was calculated by dividing the average number of bacteria on phage-treated slides by the average number of bacteria on control slides and multiplying by 100 (Rahimzadeh et al., 2022).

### 2.7 Statistical analysis

To ensure reliability, all the experiments were repeated three times. The data presented in this article are mean values with standard deviations. The analysis was carried out using MS Excel 2013, SPSS version 21 and GraphPad Prism 8.4.2. Two-Way ANOVA was employed to evaluate the effect of independent variables (two) on dependent variable. Two tailed independent sample T- test was employed when comparing two independent groups and *P* values < 0.05 were considered as statistically significant unless otherwise stated.

## 3 Results

### 3.1 Isolation and characterization of host bacteria

Uropathogenic *E. coli* isolated from the urine sample of patient with urinary tract infection was used as host bacterium for bacteriophage isolation. The host bacteria produced dark violet colored colonies with a black center and metallic green sheen around the colonies on eosine methylene blue agar, which is the characteristic feature of *E. coli*.

This strain showed resistance to amoxicillin (AML-10), bacitracin (B-10), ceftazidime (CAZ-30), clindamycin (CD-10), fusidic acid (FD-10), oxacillin (OX-1), pipracillin (30 μg), gentamicin (30 μg) while this strain was sensitive to cefixime (CFM-5) cefotaxime (CTX-30), and meropenem (10 μg).

The host bacterial strain appeared to be strong biofilm producers with an OD_570_ of 1.42 while the OD_570_ of the negative control was 0.092.

A large number of antibiotic resistance genes were identified through comprehensive genomic analysis of the whole genome of the host bacteria (Table 1). The whole genome sequence of the host bacteria designated as “*Escherichia coli* EC1” was submitted to NCBI under accession number JAUZWA000000000. This strain showed 100% similarity with *E. coli* O25b:H4-ST131, that is uropathogenic strain of *E. coli*.

**Table 1:** Antibiotics resistance mechanisms and associated genes of host bacteria that are identified by comprehensive genome analysis of whole genome of host bacteria.

**Antibiotics resistance mechanisms**	**Genes**
Antibiotics resistance genes cluster, cassette, or operon	Mar A, Mar B, Mar R
Antibiotic target in susceptible species	Ddl, Dxr, EF-G, EF-Tu, folA, Dfr, folP, gyrA, gyrB, Iso-tRNA, MurA, parC, parE/parY, rho, rpoB, rpoC, S10p, S12p
Efflux pump conferring antibiotics resistance	Mac A
Proteins altering cell wall charge conferring antibiotics resistance	GdpD, PgsA
Regulators modulating expressions of antibiotics resistance genes	GadE, H-NS

### 3.2 Bacteriophage isolation and purification

A phage against the multidrug resistant strain of uropathogenic *E. coli* EC1 was isolated from the waste water of King Abdullah Teaching Hospital using spot assay and purified by the agar double layer assay. Clear and isolated plaques with well-defined margins were observed on the host bacterial lawn, indicating the virulent nature of the isolated phage. The newly isolated phage was designated ES10.

### 3.3 Bacteriophage titer and stability determination in glycerol stock

The titer of ES10 was increased to 6.5 × 10^11^ PFU/mL and the filtrate was preserved in glycerol at a ratio of 1:1 and stability was observed for 1 year. No significant reduction in PFU of ES10 was observed over the year.

### 3.4 Characterization of ES10

#### 3.4.1 Dry lab

The size of the ES10 phage genome was 48, 315 base pairs with a GC content of 44.3% and a coding ratio of 89%. The ES10 phage genome encodes 74 proteins, 27 proteins have a known function while 47 are hypothetical. The ES10 phage did not encode antibiotic resistance genes, temperate phage life cycle genes, virulence genes, and ribosomal rRNA genes. The ES10 phage encodes the tRNA arginine gene. Forty phage promoters were found in the ES10 phage genome (Table 2).

**Table 2:** Position and the sequence of promoters present in ES10 phage genome.

**ES10 phage promoters**				
**Strand**	**Positions**	**Promoter sequence**	**Type**	**Scores**
+	(223..255)	GAGGGCTTTTTTTCGTTTGCTATATGATATAAT	Host	0.726
+	(853..860)	ACTAAATA	Host	0.667
+	(5264..5291)	TTGATTTAAATACTAAGGAAAACAAAAT	Host	0.635
+	(7715..7742)	ATGGGTTAAGATCAGTCAAATTTATAAT	Host	0.696
+	(7877..7904)	TTGCTACACCTGAAAACTCGTGTATAAT	Host	0.968
+	(8833..8861)	GTTTACCGACCTCTGACTCTTGCTATCAT	Host	0.668
+	(9214..9221)	TATAAATA	Host	0.910
+	(10730..10758)	TTGGCAGAATCAATGCTTGATGGTACAAT	Host	0.552
+	(11867..11895)	TTGTAAAAAACGAATCAAAGTAATATAAT	Host	0.957
+	(11934..11956)	TTTAAACTTTCACTAAAGGCGCT	Phage	0.599
+	(12442..12474)	TTGCCTTCCATTCTTGTTCAAAGTCTATAAAAT	Host	0.604
+	(18587..18618)	TTGACTTATCTGTTGATGGCGCAACTTACACT	Host	0.686
+	(21783..21813)	TTGACTGGAAACCGTTAGTCATTTGTATAAA	Host	0.654
+	(22971..23000)	TTTACTCTTATTATTATTATTATATATATT	Host	0.755
+	(23477..23502)	TTGAAAGCCGTTAAGATTGTTAAAGT	Host	0.907
+	(24090..24120)	TTGACCATGTGGATGTTGTTGATGGTATTGT	Host	0.643
+	(24488..24519)	TTGATAAATTTAATGATATCTTTAGATATGAT	Host	0.930
+	(27677..27703)	TGTGCTTTTTGAATCTGTATTTAAAAT	Host	0.516
+	(28726..28757)	TTGCCATTGTGTAAAGTCTCCATTTGTAAAAT	Host	0.932
+	(29296..29303)	TATAAATA	Host	0.520
+	(30301..30330)	TTAAACAATAGGTTCGATAATTGATATTAT	Host	0.885
+	(30865..30895)	TTGATAGCGAAGACCTTATCAATCATAAAAT	Host	0.980
+	(30950..30978)	TTTTACTAATGGTGTAAGACATGTATCAT	Host	0.507
+	(32272..32299)	TTGACTTGGGGTTCGTTGTTCCTACTGT	Host	0.512
+	(33996..34003)	GATAAATA	Host	0.901
+	(36814..36840)	TTGACTTCAAGTTGGAGTGATTACAAT	Host	0.978
+	(36952..36978)	ATGACGCTTGAATGGCTTCAGTATAAT	Host	0.687
+	(37066..37094)	TGTAATGGTGACAAAGCAAGTGTGATAAT	Host	0.515
+	(37604..37633)	TTGATTAGGCTTCACAGGCCTGGTTATACT	Host	0.759
+	(37957..37984)	TTGATGGCTCTTTGGTGGAATTTATTAT	Host	0.513
+	(39012..39042)	TTGACACTCTTGATAGTTCAAGCGGTAAAAA	Host	0.584
+	(43319..43346)	TTGATAACGCGTATATTCATGATACCAT	Host	0.920
+	(44118..44140)	TTAAAGTCCTGAATAAAGAAAAC	Phage	0.623
+	(45649..45675)	TTGAATAAAATTTAAGGTTTATAAAAT	Host	0.950
+	(47166..47197)	TTGACATTAAAGCGCTAAACGACAAGTACCAT	Host	0.862
+	(47484..47512)	TTGACACATCTAACCCTGACTGGTACATT	Host	0.983
+	(48161..48193)	GGGGGGGGGGGGGGGGGGGGGGGGGGGGGGGGG	Host	0.821
+	(48210..48242)	GGGGGGGGGGGGGGGGGGGGGGGGGGGGAGGGG	Host	0.641
+	(48229..48261)	GGGGGGGGGAGGGGGGGGGGGGGGGGGGGGGGG	Host	0.735
+	(48241..48273)	GGGGGGGGGGGGGGGGGGGGGGGGGGGGGGGGG	Host	0.821

**Figure 1:**
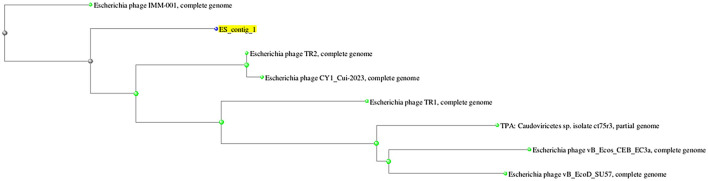
Phylogenetic tree of ES10 phage, constructed on the basis of whole genome similarity of ES10 phage with reported phages genome on NCBI.

**Figure 2:**
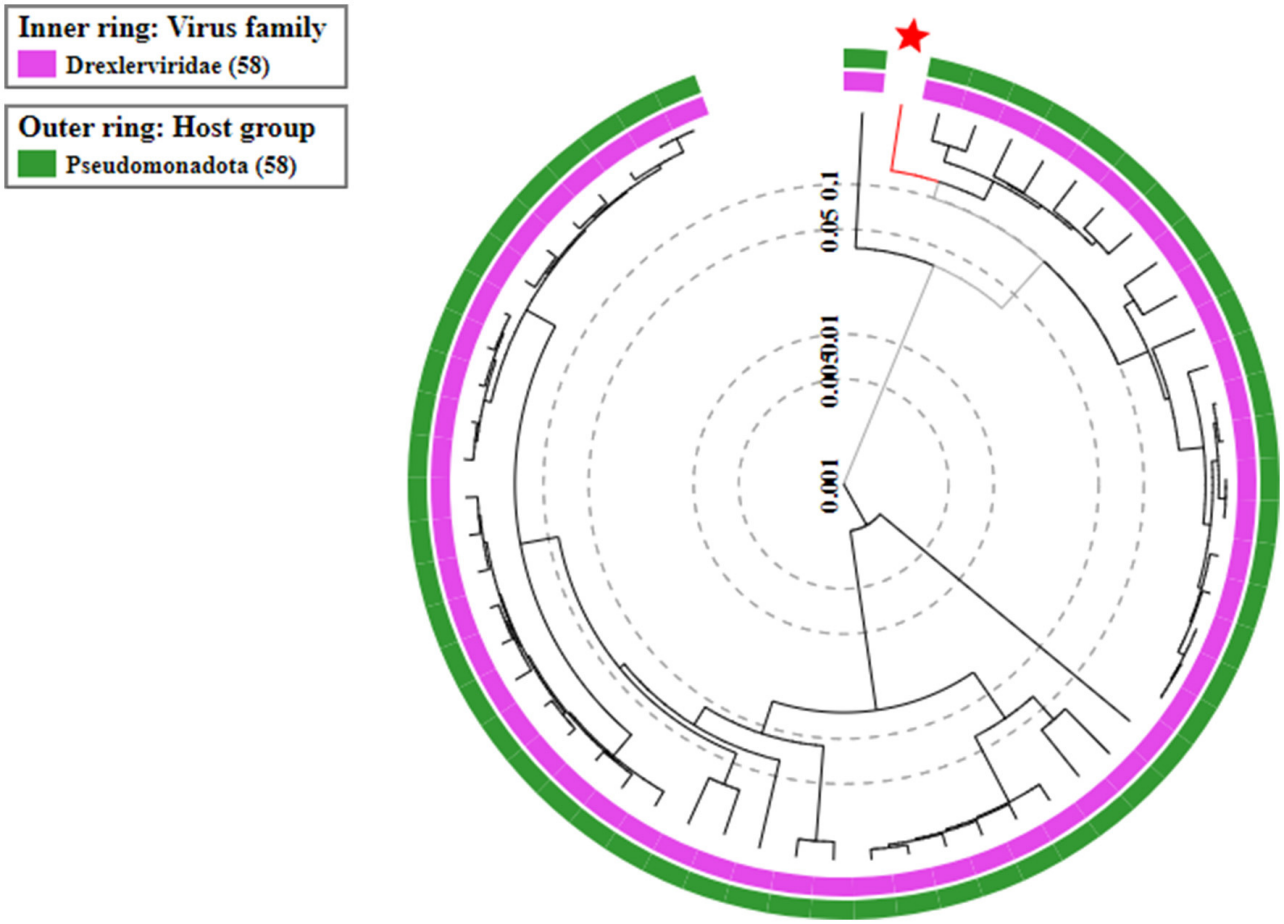
Proteomic based phylogenetic tree of ES10 phage.

On the basis of whole genome sequence of ES10 phage, phylogenetic tree was constructed (Figure 1) and ES10 phage showed similarity with the phages of family *Drexlerviridae* and the sub-family *Braunvirinae*. Based on proteomics, the ES10 phage showed maximum similarity with the phages of the *Drexlerviridae* family (Figure 2). The annotated whole genome sequence of ES10 phage is submitted to NCBI under accession number OR466728.

#### 3.4.2 Wet lab experiments

##### 3.4.2.1 Transmission electron microscopy

**Figure 3:**
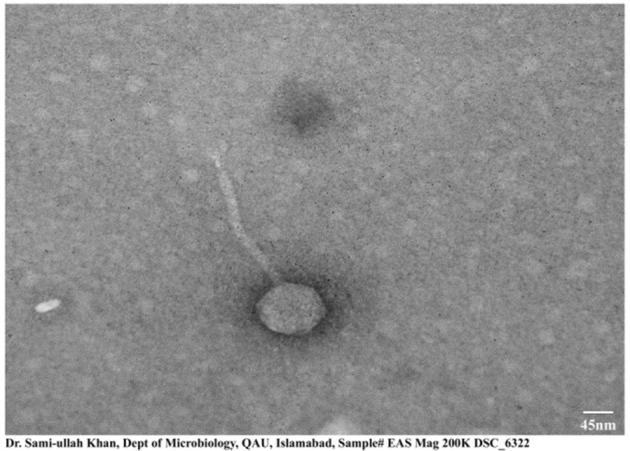
Transmission electron micrograph of ES10 phage. Scale used is of 45 nm.

The morphological structure obtained by transmission electron microscopy of ES10 showed icosahedral symmetry with a long non-contractile tail. The length of the icosahedral head was 95 ± 1.5 nm and the length of the contractile tail was 200 ± 2 nm, measured online using image J (Figure 3).

##### 3.4.2.2 Effect of different media and soft agar concentration and incubation time on plaques morphology

**Figure 4:**
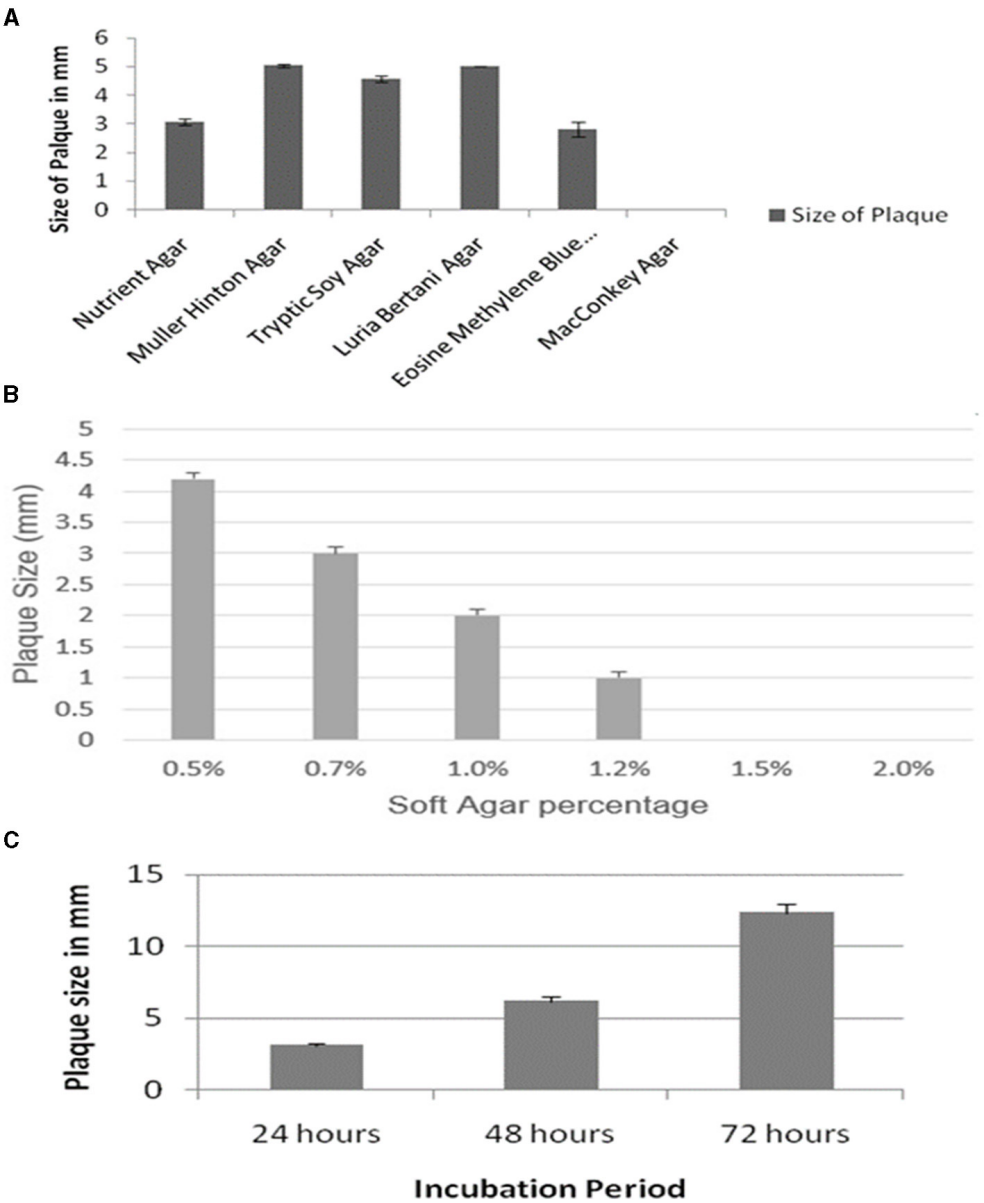
Evaluation of effect of different media on ES10 phage plaque size **(A)**, effect of different concentrations of soft gar of plaque size of ES10 phage **(B)**, and effect of incubation period on plaque size of ES10 phage **(C)**.

The effect of the composition of different bacteriological media on the plaque morphology of the ES10 phage was examined. The ES10 phage produced plaque on all media tested except MacConkey agar. The ES10 produced plaques with diameter of 5 mm on Muller Hinton agar and Luria-Bertani agar. Plaques were clear on all media except eosin methylene blue (Figure 4A).

Concentration of agar in top agar significantly affects plaque morphology. Lower concentrations of agar increase the diffusion of phages that ultimately increases plaque size. In our study, reduction in plaque size was observed with increase in agar concentration. At a concentration of 0.5% soft agar, plaque size of 4 mm was observed on nutrient agar (Figure 4B), but these plaques were turbid and diffused. Clear and well-defined plaques were observed on 0.7% soft agar.

An increase in ES10 phage plaque size was observed with increase in incubation time. After 72 h, the plaque size of 12 mm of ES10 phage was observed (Figure 4C).

##### 3.4.2.3 Thermal and pH stability

**Figure 5:**
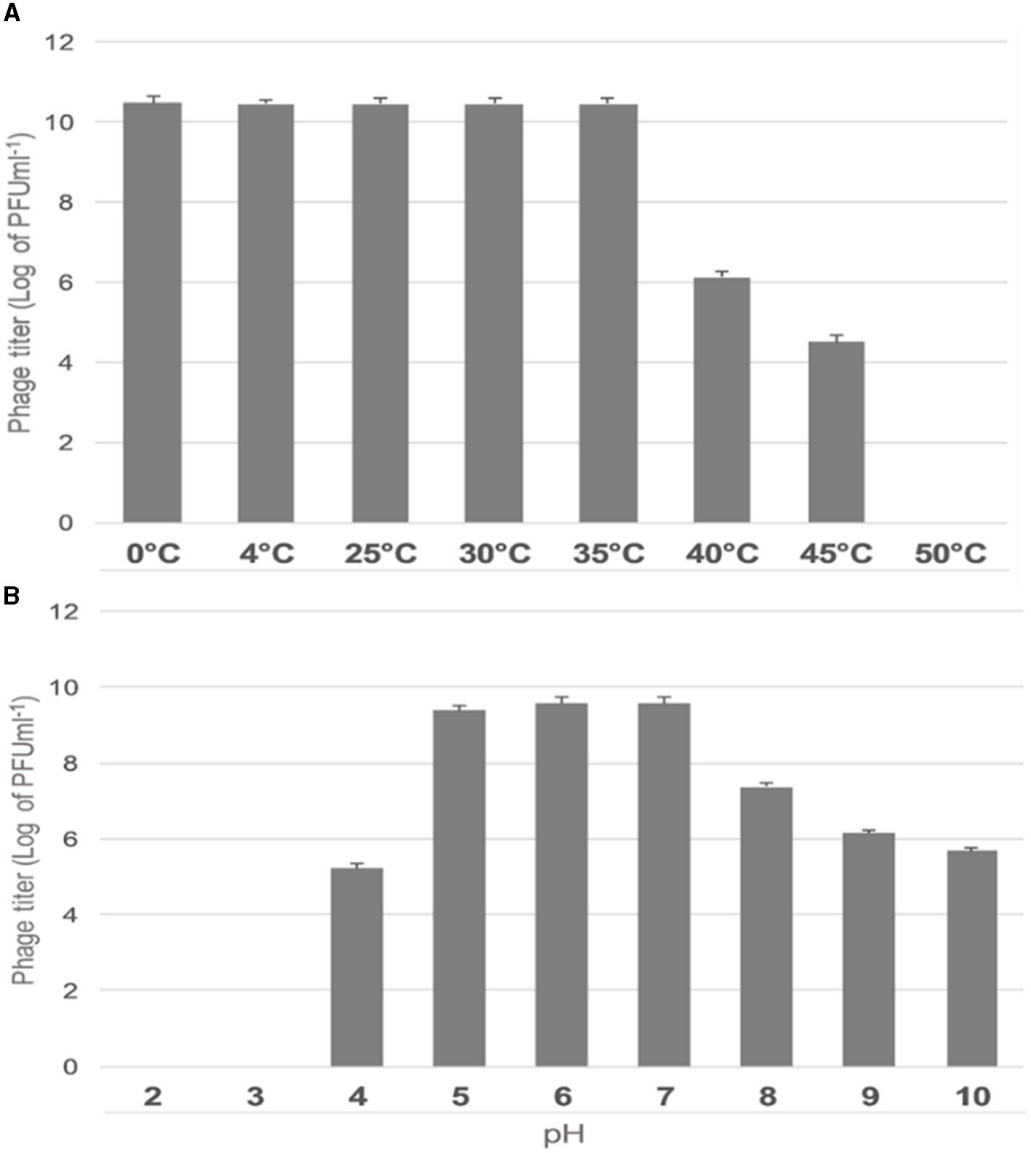
Effect of different temperature on ES10 phage stability **(A)**, effect of pH on ES10 phage stability **(B)**.

The effect of temperature on the stability of ES10 phage was evaluated by incubating ES10 phage filtrate at different temperatures and viability of ES10 phage was evaluated by double layer agar assay of dilution of ES10 phage filtrates incubated at different temperature (Figure 5A). ES10 was stable below 35°C, but a significant reduction in plaque-forming units was observed above 40°C. The stability of ES10 phage at different pH values was evaluated and the ES10 phage appeared unstable at both alkaline and acidic pH (Figure 5B). Maximum stability of ES10 phage was observed at pH5, pH 6 and pH 7.

##### 3.4.2.4 Optimal multiplicity of infection

**Figure 6:**
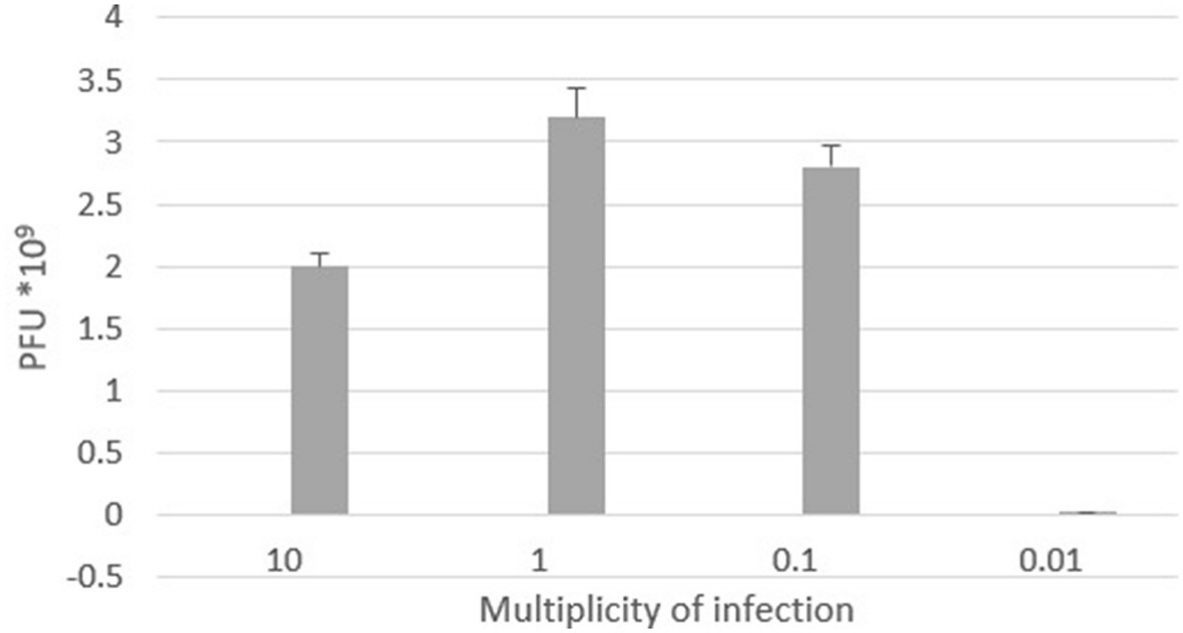
Optimal multiplicity of infection of ES10 phage.

The optimal multiplicity of infection is the number of phages required to produce the maximum number of plaque forming units after specific incubation period. In our study, the observed optimal MOI was 1 (Figure 6).

##### 3.4.2.5 One step growth

One-step growth experiment was used to evaluate latent time, burst size and number of ES10 phages required to infect a single host bacterium. In our study we used 1 mL of phage filtrate with PFU 4.6 × 10^10^/mL, and the number of host bacteria used was 3.2 × 10^9^/mL, the number of uninfected bacteria was 0, and the number of unadsorbed phages was 2.1 × 10^8^. Number of unadsorbed phages were subtracted from the total number of phages used for infection, hence the number of phages adsorbed on host bacteria were 4.58 × 10^10^/mL. Host bacterial cells infected with ES10 phage were inoculated in 100 mL of broth, hence the number of phages for infection were diluted to 4.58 × 10^8^/mL and the number of ES10 phages released after infection was 4.13 × 10^10^/mL.

The number of phages infecting single bacterium was calculated by putting above data in formula mentioned in materials and method and estimated number of phages infecting single bacterium was 14.

The burst size was calculated using the data given above.

The final PFU/ml was 4.13 × 10^10^ and the number of infected bacteria per mL was 3.2 × 10^9^. By putting this data in formula “Burst size = final PFU/number of infected bacteria,” we found that the release of phages per infected cell was 90.

**Figure 7:**
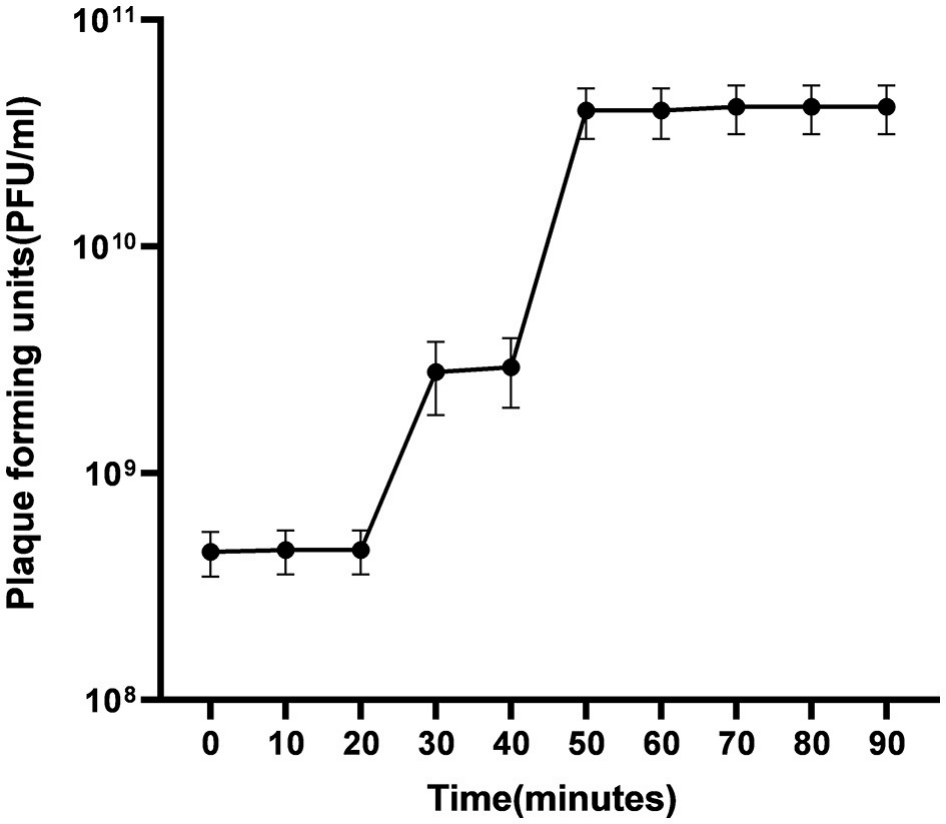
Evaluation of latent time and burst size of ES10.

The latent of ES10 phage was approximately 30 min (Figure 7).

##### 3.4.2.6 Effect of salts on the replication cycle of ES10

**Figure 8:**
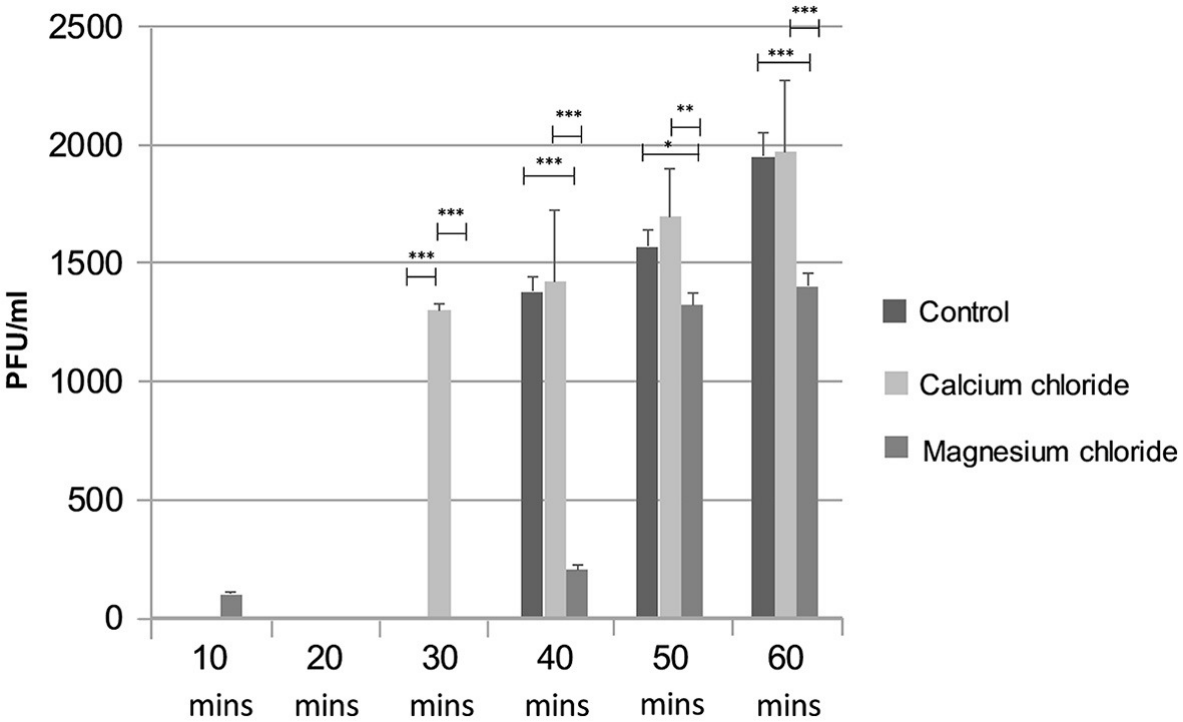
Effect of calcium chloride and magnesium chloride on replication cycle of ES10 phage. Stars are representing the difference between the groups by *p* value. P is showing the *p*-value in independent sample t-test. ^***^P < 0.001, ^**^P < 0.01, ^*^P < 0.05.

The effect of divalent cations on the ES10 replication cycle was evaluated. The addition of calcium chloride had shortened the adsorption time and latency time and resulted in early lysis of the host bacteria compared to the control, while a negative effect of magnesium on the replication cycle of ES10 phage was observed (Figure 8). Magnesium chloride had delayed the release of ES10 phage and reduced the burst size of ES10 phage.

##### 3.4.2.7 Effects of salts on turbidity reduction of host bacteria by ES10

**Figure 9:**
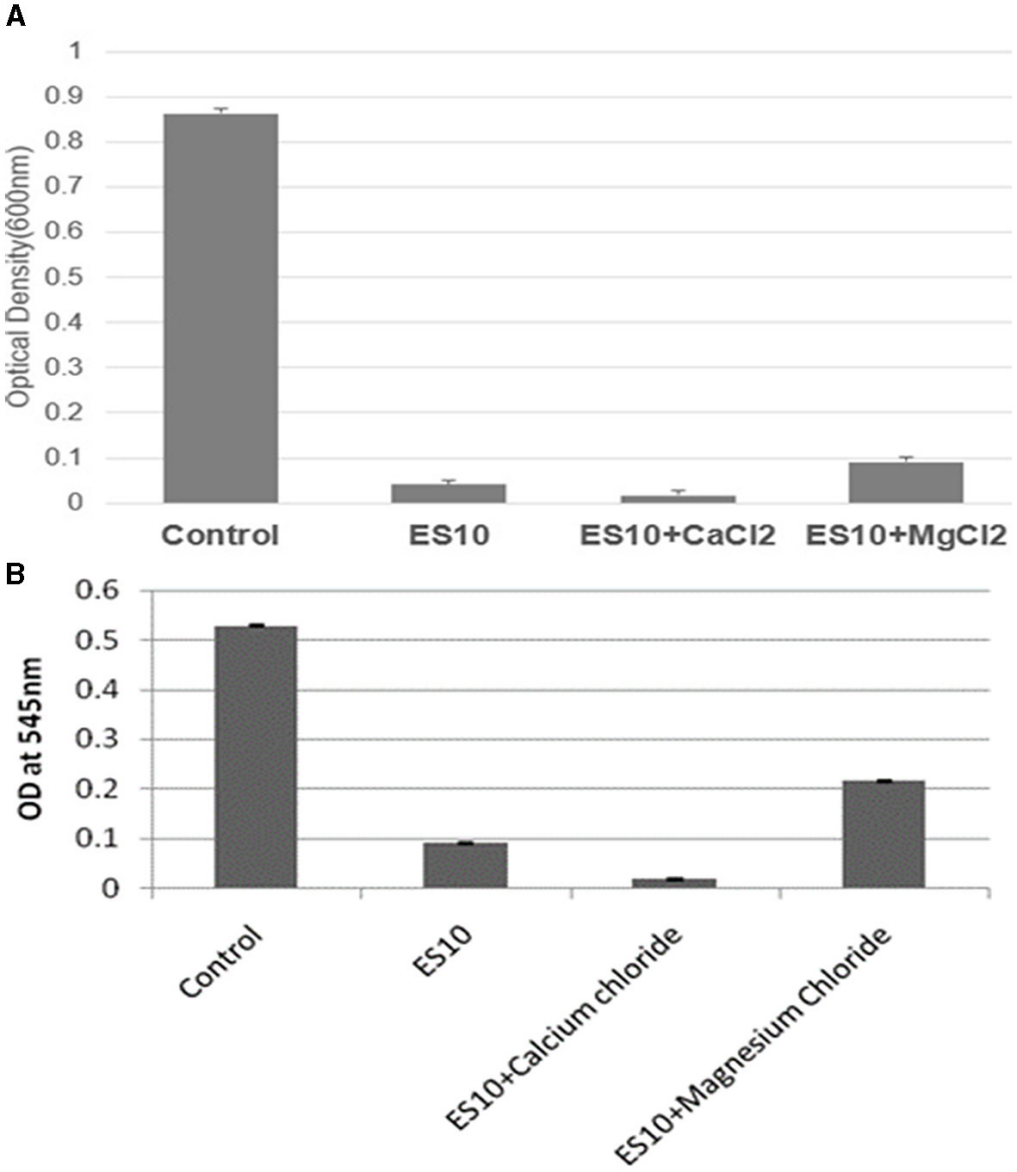
Effect of ES10 alone and ES10 in combination with calcium chloride and magnesium chloride on host bacteria turbidity reduction **(A)**, and host bacteria biofilm formation inhibition **(B)**.

The effect of ES10 phage with salts (calcium chloride and magnesium chloride) on reducing the turbidity of the host bacteria was evaluated. ES10 supplemented with calcium chloride significantly enhanced host bacteria reduction compared to ES10 alone (Figure 9A). The ES10 phage alone can inhibit the biofilm formation of the host bacteria, but the addition of calcium chloride enhanced the inhibition of biofilm formation (Figure 9B). The reduction of host bacterial turbidity and inhibition of biofilm formation by ES10 by the addition of magnesium chloride were adversely affected compared to ES10 phage alone.

##### 3.4.2.8 Effect of incubation period, temperature, pH and media on turbidity reduction of host bacteria by ES10 phage

**Figure 10:**
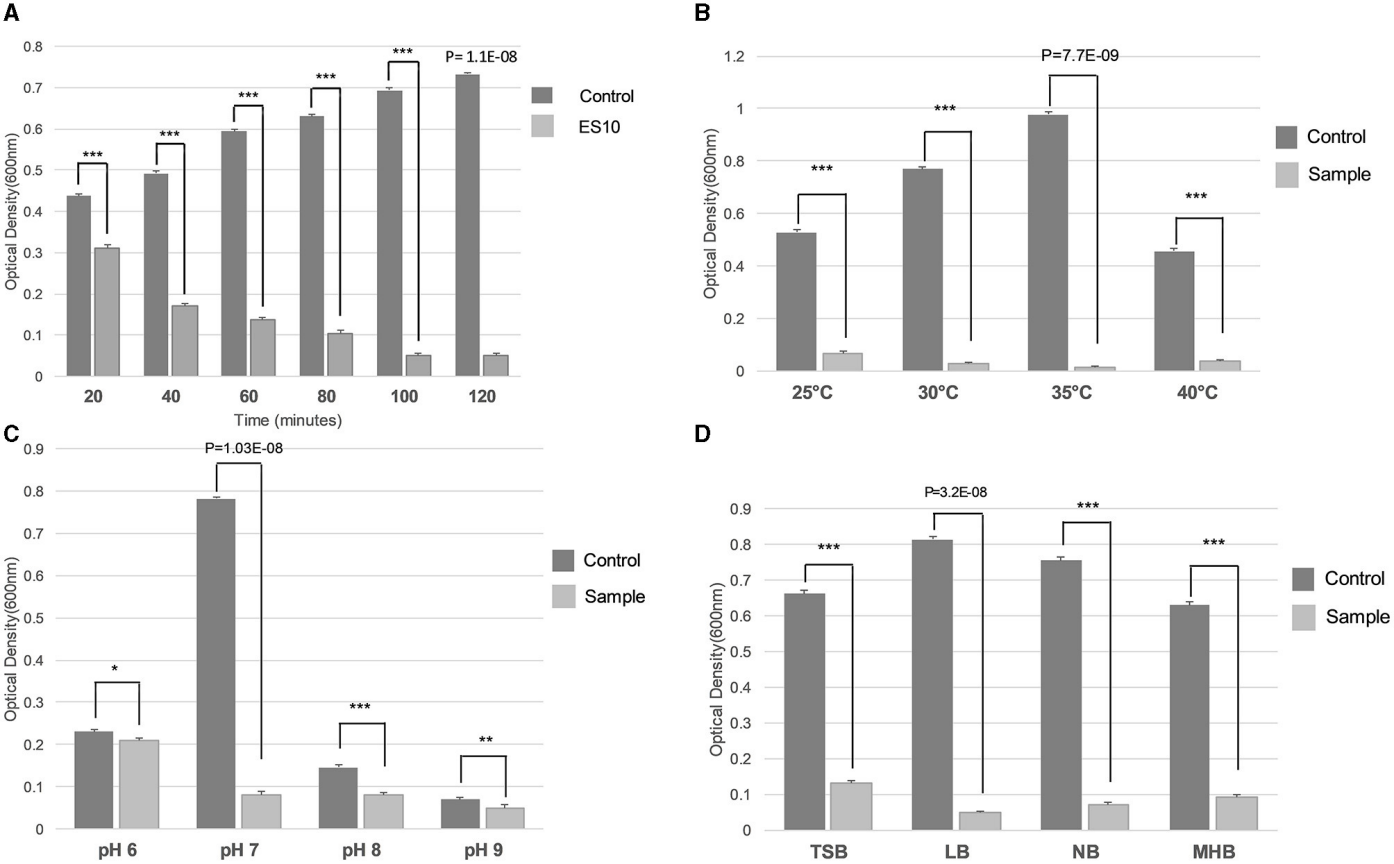
Effect of incubation period **(A)**, temperature **(B)**, pH **(C)**, and media **(D)** on turbidity reduction of host bacteria by ES10. Stars are representing the difference between the groups by *p* value. P is showing the *p*-value in independent sample t-test. ^***^P < 0.001, ^**^P < 0.01, ^*^P < 0.05.

The incubation time at which the maximum reduction of the host bacteria was achieved at an MOI of 10 was evaluated. The reduction of the host bacteria started after 40 min and the maximum reduction was observed after an incubation period of 100 min (Figure 10A). The optimal temperature for the maximum reduction of host bacteria by ES10 at an MOI of 10 for 120 min of incubation was 35°C (Figure 10B). The maximum reduction of the host bacteria by ES10 was observed at pH 7 (Figure 10C). ES10 caused a maximum reduction of host bacteria in Luria-Bertani broth compared to all media used in this study (Figure 10D).

##### 3.4.2.9 Stability of ES10 phage in human blood

To evaluate the effect of serum proteases, present in whole blood on the stability of ES10 phage, this experiment was performed. No significant phage loss was observed in the blood. The PFU of the phages in the blood after 4 h of incubation was 5.0 × 10^5^/mL compared to the control where the PFU was 5.2 × 10^5/^mL.

##### 3.4.2.10 Reduction of host bacteria by ES10 phage in human blood

A reduction in colony forming units of the host bacteria in human whole blood by the ES10 phage was observed. After 4 h of treatment of human blood contaminated with host bacteria with ES10 phage, 99.39% reduction in CFU of host bacteria was observed (Table 3). In blood, without phage treatment, increase in CFU was observed (Table 3).

**Table 3:** Effect of ES10 phage treatment on reduction in CFU of host bacteria in blood.

	**CFU/ml**
CFU of the inoculum	14,760
CFU of negative control	0
CFU of ES10 phage treated blood	90
CFU of untreated blood	397,000

##### 3.4.2.11 Fomite decontamination assay

**Figure 11:**
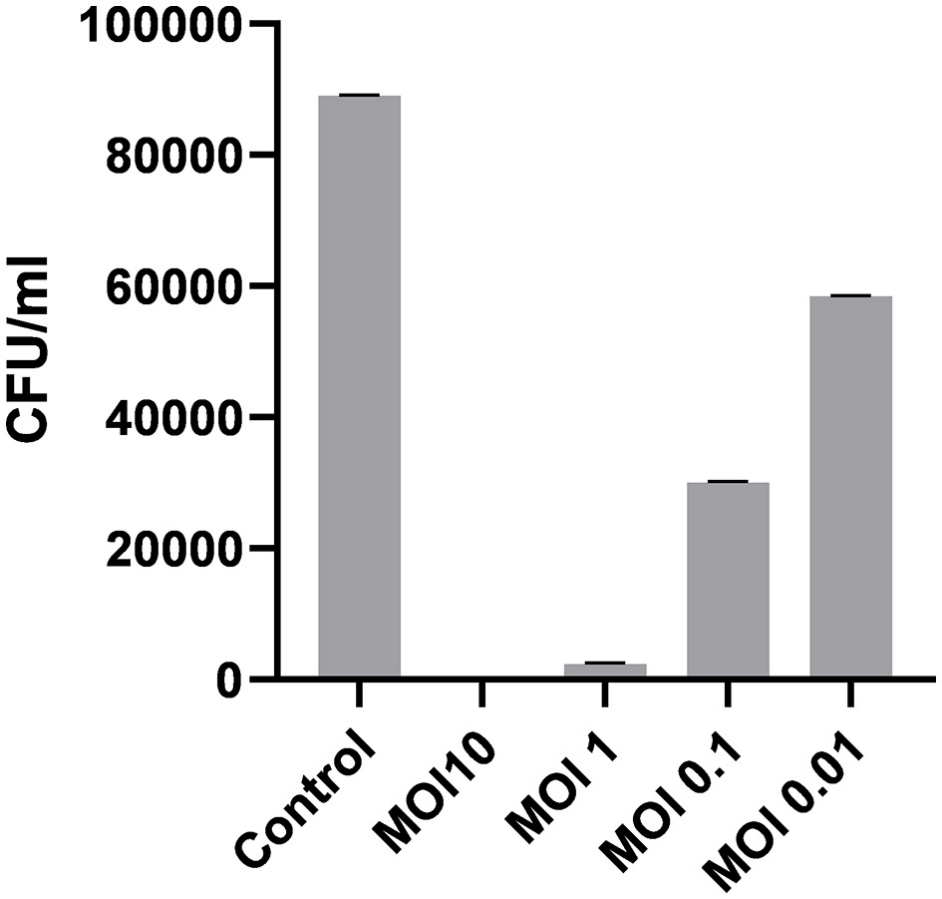
Reduction in host bacterial colony forming unit by ES10 phage on contaminated fomites.

Contaminated glass slides were used to assess the decontamination potential of ES10. The fomite decontamination assay was performed to examine the ability of the phage (at different MOIs) to eradicate *E. coli* from contaminated surfaces. The ES10 phage at MOI 10 caused a 99.84% reduction in host bacteria from contaminated glass slides after 1 h of incubation, while the ES10 phage at MOI 1 had caused a 97.3% reduction in host bacteria. The host bacteria reduction caused by the ES10 phage at MOI 0.1 was 66.2% and MOI 0.01 of the ES10 phage caused a 34.3% host bacteria reduction (Figure 11).

## 4 Discussion

Multi drug resistant pathogens are the biggest threat to public health, cause around 700,000 deaths occur per annum, worldwide, with economic loss, prolonging hospitalization, treatment failure, and increase in the cost of treatment (MacIntyre and Bui, 2017). The excessive and careless use of antibiotics in humans and animals has led to the emergence of multidrug-resistant bacteria and the post-antibiotic era is underway, in which all discovered antibiotics will no longer be effective (Nadgir et al., 2023). Antimicrobial resistance has become the second leading cause of death worldwide, and the mortality rate is estimated to reach ten million by 2050 (De Kraker et al., 2016). *E. coli* is the most common cause of urinary tract infections worldwide. The emergence of multidrug resistant strains of *E. coli* has complicated treatment options. Alternative therapies to antibiotics had to be developed within this tight timeframe. Bacteriophages are the promising alternative for treating infections caused by multidrug-resistant pathogens (Ghannad and Mohammadi, 2012). Phage therapy is considered one of the viable options for curing multi-drug resistant pathogens. The phage VB_ecoS-Golestan has been previously reported that is effective against multi-drug resistant uropathogenic *E. coli* (Yazdi et al., 2020).

This study was conducted with the aim of isolation and characterization of potent bacteriophage against multidrug-resistant strains of uropathogenic *E. coli*. The ES10 phage was isolated from hospital wastewater. Hospital sewage is the richest source of phages active against human variants of pathogenic strains (Aghaee et al., 2021). In previous study RAM-1 bacteriophage effective against multi-drug resistant strain of *Klebsiella pneumonia*-3206 was isolated from waste water sample (Shah et al., 2023).

The size of genome of ES10 phage was 48,315 base pairs long, encoding 74 proteins among them 47 are hypothetical proteins while 27 proteins have known functions. Whole genome sequence and proteomic analysis of ES10 phage showed that ES10 phage is the member of *Drexlerviridae*. MSK bacteriophage effective against *E. coli* belonging to *Drexlerviradae* family was reported previously (Khan et al., 2021). Transmission electron microscopy of ES10 phage revealed that ES10 is tailed phage with icosahedral head. The bacteriophages that belong to *Drexlerviridae* are tailed phages with icosahedral capsid.

The composition of the bacteriological media affects plaque morphology. The ES10 phage produced clear plaques of 5 mm on Muller-Hinton agar and Luria-Bertani agar. Previous study conducted on SAP1 and VPP1 reported, plaques of vivid morphology on different bacteriological media. The variation in plaque morphology on different media may be due to the composition of bacteriological media. It is believed that, the glucose production of the bacteria in Muller-Hinton agar is high enough to influence bacterial growth, which further promotes phage replication (Ramesh et al., 2019). ES10 phage did not produced plaques on MacConkey agar while plaques produced on eosine methylene blue agar were turbid. Effect of these both media on inhibition of plaques formation and turbid plaques formation might be because of presence of dyes in these media that could have effect the lytic ability of ES10 phage.

By reducing the agar concentration in top agar, enhances the phage diffusion which ultimately leads to an increases in the size of phage plaque (Abedon and Yin, 2009). In our study, an increase in phage plaque size was observed at an agar concentration of 0.5% in the top agar, but the plaques were diffused and had no clear margins, while clear plaques with defined margin were observed on 0.7% soft agar. An increase in ES10 plaque size was observed with increasing incubation time, and after 72 h of incubation, ES10 plaques were 12 mm in size. The increase in plaque size, with the increase in incubation period was due to the lysis of the host bacteria surrounding the plaque by the lytic ES10 phage.

Temperature plays an important role in the survival of bacteriophage. A higher temperature increases the latency period, while a lower temperature slows the penetration of the bacteriophage genome into the host bacteria (Gill, 2010). The ES10 phage was stable between 0–35°C and above this temperature range the viability of ES10 was compromised. Different phages behave differently to higher temperature, previous literatures had reported phages that are stable at high temperatures, their strong interactions in their structural proteins and mutations in their nucleic acid make them able to tolerate high temperature. Previous study conducted on *Klebsiella pneumoniae* phage vB_Kpn_ZC2 reported reduction in phage titer above 40°C (Fayez et al., 2023). Maximum activity of majority of bacteriophages was observed at neutral pH and slightly acidic and basic pH, while at extremities of both pH reductions in activity of majority of bacteriophages had been reported in previous literature. Maximum stability of ES10 phage was observed at pH 5, pH 6 and pH 7, reduction in PFU was observed above pH 7 and below pH 5. Activity of ES10 was completely lost at pH 2 and pH 3, and very rare bacteriophages have been reported previously that can tolerate pH 2 and pH 3, and this tolerance is due to irreversible mutations. Tolerance to lower pH is important for the utilization of phages for therapeutic purposes and lower pH of stomach is a challenge for the oral administration of bacteriophages. In previous studies, the maximum stability of WZ1 phage, belonging to *Myoviradae*, was observed at pH 7 (Jamal et al., 2015). In previous study BPA43 phage was highly stable at pH 5 and inactivation of BPA43 was observed at pH 3, 11, 12, and 13. These findings are similar to our findings.

The optimal multiplicity of infection of ES10 was 1. In determining the optimal multiplicity of infection, the plaque forming units were calculated after 24 h, hence a higher PFU was observed at MOI 1 compared to MOI 10. At a lower MOI, the number of phages is lower than the phages required to infect all bacteria, uninfected bacteria replicate, increase their numbers and act as a host for newly released phages, resulting in a high PFU. The life cycle of ES10 was assessed by a one-step growth experiment. The latency of ES10 was 30 min, the burst size was 90, and the estimated number of phages infecting a single bacterium was 14. Burst size of bacteriophages is closely related to their propagation. Proper burst size is a desirable characteristic of lytic phages to be used for therapeutic purpose, and ES10 had short latent time and large burst size. Previous study reported a latency of 20 min and a burst size of 130 for the *E. coli* phage PSH131 (Manohar et al., 2018).

Divalent cations facilitate the adsorption, penetration and multiplication of bacteriophages. Calcium ions are the most dominant factor compared to magnesium ions (Potter and Nelson, 1953). Supplementation of calcium chloride increases the burst size compared to magnesium chloride (Potter and Nelson, 1953) and to get same effect increased concentration of magnesium chloride is required (Kay, 1952). In our study, the adsorption of ES10 phage to host bacterium was enhanced and the latency was reduced by the addition of 10 mM calcium chloride. In previous studies, calcium chloride and magnesium chloride significantly enhanced the activity of phage Z (Jamal et al., 2015). In some phages, addition of magnesium chloride reduces the burst size (Rountree, 1955) and in our study magnesium chloride, thereby delayed the adsorption of ES10 to the host bacteria and prolonged the latency of ES10 phage and caused reduction in burst size. Previous study conducted on lytic *Staphylococcus aureus* bacteriophage pSa-3, reported reduction in burst size due to addition of magnesium chloride. The significant reduction in turbidity and biofilm formation of host bacteria by ES10 phages enriched with calcium chloride was observed compared to ES10 phage alone. Bacteriophages are able to kill bacteria and inhibit the formation of biofilms by means of their depolymerases and bacteriolytic enzymes. Phages invade the biofilm, destroy and disperse the biofilm (Tian et al., 2021). A previous study reported depolymerization of biofilm by bacteriophages IBEC40 and IBEC77 effective against biofilm producing uropathogenic strains of *E. coli* (Sanmukh et al., 2023). Phages are sensitive to fluctuations in physical factors, which ultimately affect their bacteriolytic activity. The ES10 phage reduced the turbidity of the host bacteria under ambient conditions.

The ES10 phage was unaffected by the proteins of freshly collected human blood, and within 4 h, a 99.34% reduction of the host bacteria was caused by the ES10 phage in human blood, proving the stability and activity of ES10 in human blood. Previous study conducted on propagation of SA-phage in human blood, serum and plasma had reported that, blood composition had inhibited SA-phage replication (Frati et al., 2019), this study is contradicting our findings.

The fomites decontamination potential of ES10 observed in this study was approximately 99.84% at MOI 10. A previous study published in 2015 by Jensen et al., reported the fomites decontamination potential of phages from methicillin-resistant *S. aureus* (Jensen et al., 2015).

In this study, all the experiments were conducted thrice to ensure the reliability of presented data and graphs are plotted on the mean values. Standard deviation was employed for the data interpretation ES10 phage morphology, optimal MOI, latent time, burst size, phage particles per bacterium, effect of salts on ES10 phage replication and efficiency, stability and activity of ES10 in human blood and efficacy of ES10 on eradication of host bacterium from contaminated fomites. While the interpretation of data of turbidity reduction experiments, two tailed sample *T-*test was employed, to compare the mean of OD reduction of control with the mean of OD reduction of ES10 phage treated host bacterium and to determine the significant difference.

## 5 Conclusion

Uropathogenic multi-drug resistant *E. coli* is the main cause of urinary tract infections. Antibiotics are no longer effective against resistant *E. coli* strains, complicating this infection and leading to higher mortality rates and health care costs. Since the last decade, numerous antibiotics have been approved but parallel to the discovery of antibiotics there has also been the emergence of multi-drug resistance pathogens. In this regard, bacteriophages appear to be a promising alternative to antibiotics against multi-drug resistant pathogens. In this study, the isolated phage, designated ES10, carries no virulence genes, no antibiotics resistance genes and temperate lifestyle gene. The ES10 phage has a short replication cycle and effectively eliminates bacteria from infected human blood and contaminated glass slides. ES10 is a potent bacteriolytic phage and can be considered for antibacterial therapy after studying it's *in vivo* efficacy.

## Data availability statement

The datasets presented in this study can be found in online repositories. The names of the repository/repositories and accession number(s) can be found in the article/supplementary material.

## Author contributions

AN: Conceptualization, Funding acquisition, Investigation, Methodology, Writing – original draft, Writing – review & editing. SZ: Formal analysis, Investigation, Writing – review & editing. AHA: Formal analysis, Writing – review & editing. NAK: Formal analysis, Software, Writing – review & editing. MS: Formal analysis, Investigation, Writing – review & editing. AM: Formal analysis, Investigation, Writing – review & editing. MB: Formal analysis, Resources, Writing – review & editing. AAS: Formal analysis, Funding acquisition, Resources, Supervision, Writing – review & editing. SK: Conceptualization, Funding acquisition, Resources, Supervision, Writing – review & editing.
